# Geographical mobility of UK trainee doctors, from family home to first job: a national cohort study

**DOI:** 10.1186/s12909-018-1414-9

**Published:** 2018-12-20

**Authors:** Ben Kumwenda, Jennifer A. Cleland, Gordon J. Prescott, Kim A. Walker, Peter W. Johnston

**Affiliations:** 1 Institute of Education for Medical and Dental Sciences, School of Medicine, Medical Sciences & Nutrition, University of Aberdeen, Aberdeen, UK; 2 Medical Statistics Team, School of Medicine, Medical Sciences & Nutrition, University of Aberdeen, Aberdeen, UK; 3 NHS Education for Scotland and UK Foundation Programme Office, Forestgrove House, Foresterhill, Aberdeen, UK; 4 NHS Grampian, The Scotland Deanery, Aberdeen, UK

## Abstract

**Background:**

The UK faces geographical variation in the recruitment of doctors. Understanding where medical graduates choose to go for training is important because doctors are more likely to consider practicing in areas where they completed postgraduate training. The wider literature also suggests that there is a relationship between origin and background, and where doctors wish to train/work. Thus, the purpose of this paper is to investigate the geographical mobility of UK medical graduates from different socio-economic groups in terms of where they wish to spend their first years of postgraduate training.

**Methods:**

This was an observational study of Foundation Programme (FP) doctors who graduated from 33 UK medical schools between 2012 and 2014. Data was accessed via the UK medical education database (UKMED: https://www.ukmed.ac.uk/). Chi-square tests were used to examine the relationships between doctor’s sociodemographic characteristics and the dependent variable, average driving time from parental home to foundation school/region. Generalised Linear Mixed Models (GLMM) were used to estimate the effects of those factors in combination against the outcome measure.

**Results:**

The majority of doctors prefer to train at foundation schools that are reasonably close to the family home. Those who attended state-funded schools, from non-white ethnic groups and/or from lower socio-economic groups were significantly more likely to choose foundation schools nearer their parental home. Doctors from disadvantaged backgrounds (as determined by entitlement to free school meals, OR = 1.29, *p* = 0.003 and no parental degree, OR = 1.34, *p* < 0.001) were associated with higher odds of selecting a foundation schools that were closer to parental home.

**Conclusion:**

The data suggests that recruiting medical students from lower socioeconomic groups and those who originate from under-recruiting areas may be at least part of the solution to filling training posts in these areas. This has obvious implications for the widening access agenda, and equitable distribution of health services.

## Background

Imbalanced distribution of health care workforce remains a major concern worldwide [1–4]. Certain regions and localities face chronic under-recruitment, while others have more applicants than there are training posts and jobs. A growing body of literature has examined the reasons why some areas are unpopular, while others are over-subscribed [5–9] Most of this research uses the push-pull framework to identify factors that motivate individuals to move to certain areas [5–10]. The pull factors typically include opportunities for professional training, offers of higher wages, and better employment opportunities [9–11]. While the influence of socio-economic factors and career advancement opportunities play a key role in determining where individuals chose to work, personal factors, such as desire to train/work somewhere near family and friends are also known to be very important [9, 12, 13] yet the implications of these preferences are rarely examined in detail.

In UK, there have been policy drives to address the shortage of doctors in certain geographical areas [7, 14]. These policies are often supported by the emerging evidence that doctors tend to go back and work in areas where their training was undertaken [6, 7, 15–17]. Yet this is an issue for several reasons. Firstly, medical schools are mal-distributed around the UK. For example, East of England has 7.4% of yearly intake of medical students and 9.3% of the UK population, while London area has 26.7% of medical students and 13.4% of UK population [18, 19]. Second, because of where UK medical schools are located, for some people there is no medical school that they could attend while staying at home.

This is important to consider as the wider research suggests that people from lower socioeconomic groups are more likely to study and work nearer home [20, 21]. But unlike other disciplines, very little is known about the social background of students who go to nearby medical schools and train elsewhere, or vice versa. Few studies that have examined the link between home origin and place of medical training did not look at the socio-demographic composition of the different groups [6, 16]. Other studies that have looked at the subject are not specific to medicine [22–25], or they are based on qualitative methodologies, which focused on a restricted temporal window and did not account fully for the original residence of the student (often the family home) [21, 26, 27].

Increased access to higher education gives students the flexibility of choosing where to go and what to study. Nearly 70% of the 2.4 million students in UK’s higher education lived away from home during a period of their study [28, 29]. The perceived advantages of studying away from home include greater life experience which could unlock more career prospects. However, distance from home to higher education remains an issue for certain groups of people. A 2005 study by Patiniotis and Holdworth reported that 22.7% of the 3262 undergraduate students they surveyed were living in the parental home whilst at university, primarily for financial reasons [21]. Other qualitative studies have cited the cost of living away from home, and the cost incurred when commuting as being the reasons why some students live at home while at university [20, 27, 30]. In general, students from low social class backgrounds are more likely to worry about studying away from home than the socially advantaged students.

Addressing the continuing problem of doctor mal-distribution requires further understanding of the migration patterns of medical graduates and insight into factors that influence their movement. To address the first of these questions, we are looking at the question of doctors progressing from medical school to first training post, the foundation programme (FP). The nature of the FP application process means we know applicant preferences for where they want to work, and this can be compared to family postcode on application to medical school. The UK foundation training provides a generic professional training that bridges the transition from medical school into specialist training. This training pathway is flexible in terms of movement across the four UK countries. Applicants to the foundation school are allocated based on their scores during the national application process and expressed in order of preference (see later) [31].

Thus, the purpose of this paper is to investigate the geographical mobility of UK medical graduates in terms of where they wish to spend their first years of postgraduate training, and how this relates to where they attended medical school and parental home. To further inform the discussion, we also looked at the relationship between socio-economic variables and proximity between home and training locality. Although the data is specific to the UK environment, we believe our methodology and outcomes will be of interest to medical education researchers beyond the UK.

## Methods

### Study context

Our context is the UK’s postgraduate medical training pathway. UK medical students spend between four and 6 years at medical school before they enter foundation training, the generic two-year training programme (the Foundation Programme: FP) which bridges the gap between finishing medical school and becoming eligible to apply for specialty (residency) training. Once students graduate from medical school they can apply to train at any of the 21 foundation schools throughout the UK. At the end of the first year of the FP, doctors who have successfully achieved their competencies gain full registration with the General Medical Council (GMC). Following this, the second year of the FP (F2) is the first opportunity for doctors to apply for a specialty training post.

### Data description

Our sample included students who graduated from 33 UK medical schools between 2012 and 2014, and applied for allocation to the Foundation Programme (FP), the generic 2-year postgraduate training which immediately follows medical school in the UK. This represents a 100% inclusion rate of all medical schools. The focus of this analysis is trainees who accepted an offer at their first preference of foundation school.

We used linked individual-level data from the UK medical education database (UKMED: https://www.ukmed.ac.uk/) as the basis for this study. UKMED allows the analysis of data from a number of sources, including medical school admissions, assessment and postgraduate training [32]. UKMED also contains self-declared demographic data such as age, gender and ethnicity. Students’ ethnicity is grouped as either White (the majority ethnic group) or from minority ethnic groups such as Asian, Black, or mixed race. The UKMED also contains variables that relate to academic performance and socio-economic status –with the latter used in previous research examining factors that influence educational achievement of students from different backgrounds, particularly in terms of widening participation [33–36]. These socio-economic variables include: parental postcode at the time the student applied to medical school; parental occupation (derived from National Statistics Socioeconomic Classification); income support; entitlement to free school meals; POLAR, which is an indicator of the participation of young people in higher education by geographic area; Index of Multiple Deprivation (IMD), which is an area measure of socioeconomic status routinely used in UK education and health services research; type of school (state-funded or independent); and parental education. We also included place of medical qualification in the analysis (UK country: England, Scotland, Wales and Northern Ireland).

Twenty-one regional foundation schools offered the FP at the time of the study. Given the unequal distribution of medical schools and foundation schools [8, 19], those in England were divided into three groups for analysis; London Area, South of England and the Rest of England.

Access to the data was limited to specific members of the research team via a safe haven (to ensure adherence to the highest standards of security, governance and confidentiality when storing, handling and analysing identifiable data). Ethics approval was not required because the focus of this study was a secondary analysis of anonymised data [32]. All the data analyses were done using IBM SPSS Statistics for Windows, Version 24 (IBM Corp., Armonk, NY, USA).

### Outcome data

The process of appending a spatial reference to a foundation school imposes a constraint on the research design because these are “virtual” units, spanning at times quite large geographical areas. Moreover, the FP consists of six 4-month blocks of different specialties, which can mean rotating through different hospitals and general practices, all within the same foundation school but potentially in different localities within the foundation school geographical area. While bearing this in mind, we used the postcode of the administrative office as a centroid of each foundation school. We then used a web-based distance calculator (https://www.doogal.co.uk/drivingdistances.php) to measure the travel-time between parental home and foundation school. This customised application uses Geographical Information Systems (GIS) technique to generate driving, cycling or walking distances between two postcodes or latitudes/longitudes. It uses Google Maps to determine the fastest driving route, which is then converted into actual driving time. By combining parental home and foundation school administrative office postcodes, we were able to generate data on proximity between home and training locality.

### Statistical analysis

Because of the skewed nature of the outcome variable, average driving time to foundation school was expressed as a three-category ordinal measure. The following outcome values were assigned: 1 for the travel time that was less than 2 h; 2 for the travel time that was between two and 4 h, and 3 for all the distance travelled for more than 4 h. Chi-square tests were used to examine the bivariate relationships between trainees’ socio-demographic characteristics and the outcome variable, average driving time to foundation school. Statistical significance was set a priori at *p* < 0.05. We also calculated a net gain or loss of trainees moving from one region of medical school to another region of foundation school. A net gain or loss percentage, out of the total number of trainees in the corresponding region is also presented.

After assessing the differences, a Generalised Linear Mixed Model [37] (GLMM) was used to estimate the effect of the factors that were statistically significant against the outcome measure. The GLMM uses multilevel modelling technique to allow us account for the grouped structure of the data – with individuals nested within different UK countries. A key feature of the model is that the probability of an event occurring is defined in terms of cumulative probabilities, and not just the odds of individual levels of dependent variable (the ordinal regression models). Adopting the notations of Heck, Thomas and Tabata [37], the model is denoted by the equation:

$$ {\eta}_{iC}=\mathit{\log}\left(\frac{\pi_{iC}}{1-{\pi}_{iC}}\right)={\theta}_C+{\beta}_1+{\beta}_2\dots {\beta}_4 $$

Where, *η*_*iC*_is the latent variable that predicts the log odds for individual *i* in group *c* being in the lowest category of the explanatory variable*.* θ_C_ is the increase in model threshold, while *β*_1_…*β*_4_ represents the slope coefficients across categories.

## Results

### Univariate analysis

Applicants rank their choice of the Foundation School in order of preference. This analysis was of 5689 (67.2%) trainees who accepted an offer at their first choice of foundation school (out of a total of 8467 UK only trainees who accepted an offer at any foundation school). Explanatory variables were described by the outcome measure, average driving-time split into three categories.

Table 1 gives an overview of the mean driving time from parental home to administrative office of the Foundation School by sociodemographic marker. The first choice of foundation school with less than two-hours driving time from the parental home was selected by 61.9% of the trainees, between two and 4 h by slightly above quarter (26.4%) and the remaining 11.7% chose a foundation school that was more than 4 h from the parental home. The following groups were significantly more likely to choose a foundation school that was nearer their parental home: those who attended state-funded schools; from lower socio-economic groups as defined by parental occupation, entitlement to free school meals, or being first in the family to go to university; trainees from areas that send fewer students to higher education (POLAR); trainees from Asian and other minority groups. The other markers of individual deprivation, specifically index of multiple deprivation (IMD) and receiving income support, had no significant relationships with distance to preferred foundation school.

**Table 1:** Driving time from parental postcode to Unit of Application (UoA)

	Overall sample distribution		Less than 2 h (*n* = 3519, 61.9%)		Between 2 and 4 h (*n* = 1503, 26.4%)		More than 4 h (*n* = 667, 11.7%)		*p*-value
	n	%	n	Row %	n	Row %	n	Row %	
Gender									
Female	3318	58.3	2039	61.5	889	26.8	390	11.8	0.728
Male	2371	41.7	1480	62.4	614	25.9	277	11.7	
Age Category									
School Leaver (17–20)	5041	88.6	3092	61.3	1353	26.8	596	11.8	0.072
Mature (21+)	648	11.4	427	65.9	150	23.1	71	11.0	
Highest qualification on entry to medical school									
Graduate	471	8.3	314	66.7	105	22.3	52	11.0	0.069
Non-Grad	5218	91.7	3205	61.4	1398	26.8	615	11.8	
Type of secondary school attended									
State-funded	3932	71.1	2469	62.8	988	25.1	475	12.1	0.001
Fee paying school	1597	28.9	947	59.3	479	30.0	171	10.7	
Parental Occupation									
I-Managerial and professional occupations	4128	90.1	2475	60.0	1140	27.6	513	12.4	< 0.001
Other occupations (NS-SEC II – V)	456	9.9	321	70.4	92	20.2	43	9.4	
Free School Meal									
Yes	380	7.3	281	73.9	61	16.1	38	10.0	< 0.001
No	4858	92.7	2968	61.1	1321	27.2	569	11.7	
Income Support									
Yes	692	13.8	444	64.2	171	24.7	77	11.1	0.395
No	4308	86.2	2650	61.5	1161	26.9	497	11.5	
Parent Education (University degree)									
Yes	3849	71.1	2289	59.5	1090	28.3	470	12.2	< 0.001
No	1564	28.9	1075	68.7	338	21.6	151	9.7	
(Neighbourhood) of participation of higher education areas (POLAR 2&3)									
Low Participation	287	5.1	192	66.9	80	27.9	15	5.2	0.003
High Participation	5369	94.9	3320	62.1	1502	26.6	642	11.4	
Ethnicity									
Asian or Asian British	921	16.2	728	79.0	155	16.8	38	4.1	< 0.001
Black or Black British	96	1.7	59	61.5	28	29.2	9	9.4	
Mixed	209	3.7	120	57.4	68	32.5	21	10.0	
Other Ethnic Groups	105	1.8	84	80.0	16	15.2	5	4.8	
White	4349	76.6	2521	58.0	1234	28.4	594	13.7	
Domicile									
England	4340	76.6	2746	63.3	1261	29.1	333	7.7	< 0.001
Northern Ireland	374	6.6	236	63.1	0	0.0	138	36.9	
Scotland	706	12.5	415	58.8	152	21.5	139	19.7	
Wales	245	4.3	122	49.8	90	36.7	33	13.5	
Foundation School [Region]									
London Area	1888	33.2	1259	66.7	452	23.9	177	9.4	< 0.001
Northern Ireland	247	4.3	236	95.5	0	0	11	4.5	
Rest of England	1785	31.4	1055	59.1	562	31.5	168	9.4	
Scotland	713	12.5	415	58.2	135	18.9	163	22.9	
South of England	836	14.7	447	53.5	258	30.9	131	15.7	
Wales	220	3.9	107	48.6	96	43.6	17	7.7	

The results are based on the assumption that the applicants lived at the address given as their parental postcode

Table 2 shows the movement pattern of trainees between region of medical qualification and foundation school location. The result of inter-region movement is expressed as a net gain or loss. The shaded cells indicate the proportion of trainees who went to foundation schools that were in the same region of the medical school they had graduated from. The table shows that the London Area, Northern Ireland and South of England had net gains; while Scotland, Wales and the Rest of England had net losses. The biggest net gain was noted in London Area (53.4%), while the biggest net loss was observed in the region classified as Rest of England (− 23.0%). A comparison between UKs devolved nations shows that Wales (− 20.3%) had the highest proportion of graduates who moved out of the region compared to Scotland (− 15.2%) and Northern Ireland (+ 4.7%). We also extended the analysis to look at individuals who trained in a country of their domicile or moved to different country. In summary, 74% (529/713) of medical graduates who remained in Scotland had Scottish home postcodes, compared to 48% (105/220) of trainees in Wales who had a local home postcode. Of the 247 medical graduates who remained in Northern Ireland, 96% (236/247) had a Northern Ireland parental post code. And 91% (4108/4509) of trainees in England had an English parental postcode.

**Table 2:** Migration from Medical School to Foundation School by Location

Location of School of Medical Qualification	Location of Foundation School, n (%)												Trainees who left their medical school location, n (%)	Net Gain or Loss (%)^a^
	London Area (*n* = 1888)		Northern Ireland (*n* = 247)		Rest of England (*n* = 1785)		Scotland (*n* = 713)		South of England (*n* = 836)		Wales (*n* = 220)			
	N	%	N	%	N	%	N	%	N	%	N	%		
London Area (*n* = 1231)	879	71.4	2	0.2	140	11.4	6	0.5	197	16.0	7	0.6	352 (28.6)	+ 657 (+ 53.4)
Northern Ireland (*n* = 236)	4	1.7	212	89.8	6	2.5	10	4.2	3	1.3	1	0.4	24 (10.2)	+ 11 (+ 4.7)
Rest of England (*n* = 2319)	618	26.6	10	0.4	1421	61.3	58	2.5	191	8.2	21	0.9	898 (38.7)	− 534 (−23.0)
Scotland (*n* = 841)	87	10.3	15	1.8	60	7.1	613	72.9	63	7.5	3	0.4	228 (27.1)	−128 (−15.2)
South of England (*n* = 786)	276	35.1	8	1.0	136	17.3	23	2.9	328	41.7	15	1.9	458 (58.2)	+ 50 (+ 6.4)
Wales (*n* = 276)	24	8.7	0	0.0	22	8.0	3	1.1	54	19.6	173	62.7	103 (37.3)	−56 (−20.3)

^a^A net gain or loss refers to the sum of a gain and a loss of trainees in each (medical school) region; the percentage indicates the net gain or loss divided by the total number of trainees

**Fig. 1:**
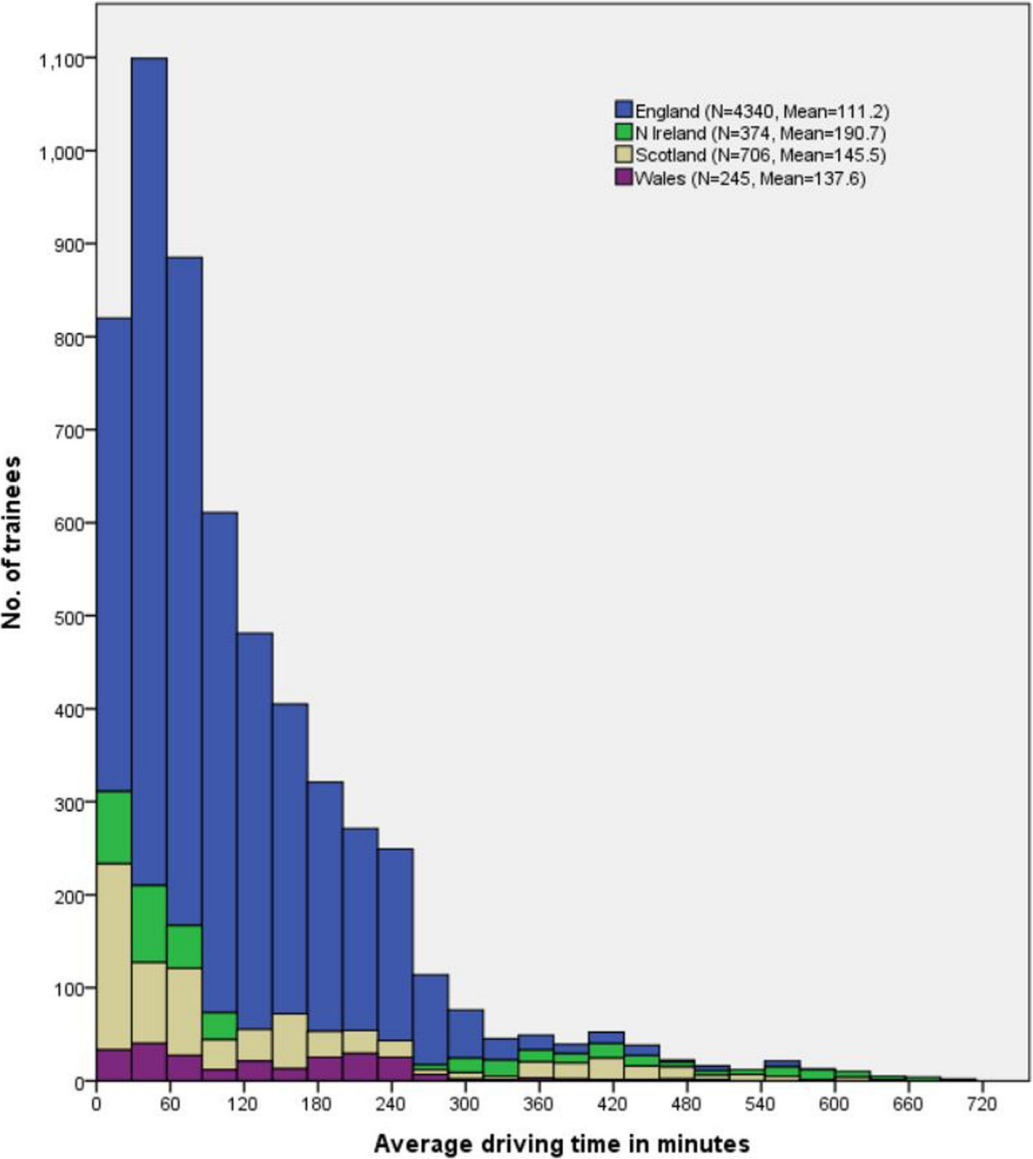
Trainees’ travel time from parental home to foundation school

Figure 1 shows the distribution of travel time in minutes for all of these trainees and split by UK country of parental postcode. As we might expect, given that Northern Ireland has only one foundation school and is geographically separate from the rest of the UK, no trainees from Northern Ireland have category two of the outcome measure (between two and 4 h) of travel time between their foundation school and home postcode. Conversely, since we are using the estimated travel time by road network (rather than by air), trainees from Northern Ireland who attend foundation schools in England, Scotland or Wales are more than 4 h away from home.

### Multilevel Modelling - Generalised Linear Mixed-effects Models

Table 1 implies that trainees from Wales have the shorter travel time compared with those from other countries, but this could be an artefact of geographical accessibility. To control for this possibility, we used Generalised Linear Mixed-effects Models (GLMM) in which we included ‘UK country’ as a random-effect term to account for the non-independence effect that may arise from the location of the country of parental postcode. To avoid overfitting, only variables that were statistically significant at *p* < 0.05 in the chi-square tests were fitted to the GLMM. We present two sets of models: one that assessed the variation in individual-level factors (unadjusted) and the other that assessed the effect of all other variables at country level (adjusted).

The model is summarised in Table 3. The shortest driving time (less than 2 h driving time) was taken as the reference category. The result shows the outcome is ordinal, the probability distribution is multinomial, and the link is cumulative logit. As the level 2 (within-country) results are consistent with the previous level 1 (individual-level) model, we will concentrate on the effects of the level 2 results. The result confirms that coming from disadvantaged backgrounds (as determined by free school meal, OR = 0.776, *p* = 0.003; parental education, OR = 1.344, *p* < 0.001 and POLAR, OR = 1.225, *p* = 0.041) influences trainees’ odds of selecting foundation schools closer home. More specifically, holding the other factors (and random-variable component) in the model constant, the expected odds of attending a foundation school that has a less than 2 h driving time from the parental home is increased by a factor of 1.29 (or increased by 29%) for trainees who at some point were recipient of free school meals versus those who were not. Parental education was also found to be of importance, trainees whose parents were educated to degree level, increased the expected odds of selecting a foundation school more than 2 h of driving time by 1.34 times (or increased by 34%). Trainees of Asian background had 2.4 times (CI [1.795–3.211], *p* < 0.001) the odds of choosing a foundation school that was closer home than trainees from Caucasian backgrounds.

**Table 3:** Results from Generalised Linear Mixed Models (GLMM), where the effect of UK Country is included in the model

Predictor Variable	Unadjusted Odds Ratio				Adjusted Odds Ratio			
	Odds Ratio	Sig	95% Confidence Interval for Exp(coefficient)		Odds Ratio	Sig	95% Confidence Interval for Exp(coefficient)	
			Lower	Upper			Lower	Upper
Threshold for driving time								
Over 4 h	0.112	< 0.001	0.063	0.198	0.132	< 0.001	0.077	0.227
Between 2 to 4 h	0.536	0.02	0.317	0.908	0.667	0.125	0.398	1.119
2 h or less*								
Free School Meal								
Yes	1.644	< 0.001	1.331	2.030	1.289	0.003	1.092	1.520
No*								
Parent Degree								
No	1.435	< 0.001	1.297	1.589	1.344	< 0.001	1.236	1.462
Yes*								
POLAR								
Low Participation	1.337	0.001	1.123	1.593	1.225	0.041	1.009	1.488
Other*								
Ethnicity								
Asian or British Asian	2.556	< 0.001	2.024	3.229	2.401	< 0.001	1.795	3.211
Black or Black British	1.157	0.472	0.777	1.722	1.227	0.434	0.735	2.050
Mixed	0.997	0.979	0.819	1.214	0.981	0.868	0.785	1.227
Other Ethnic Groups	2.696	< 0.001	1.912	3.802	2.999	< 0.001	2.102	4.277
White*								
Random Effect								
Var (Intercept)								
Estimate	0.500	0.134	0.135	1.845	0.448	0.138	0.12	1.682

* denotes the reference category

## Discussion

Medicine, in principle, is a profession that permits high levels of geographical mobility [16]. However, lack of largescale linked geospatial data has prevented researchers from examining doctors’ mobility patterns in detail to date [32, 38]. Indeed, to the best of our knowledge, this is the first study to use travel time to analyse the relationship between distance from parental home and location of postgraduate training school. We identified that the majority of medical graduates prefer to do their foundation training relatively close to the family home. However, those who were at some point recipients of free school meals, whose parents are not educated to degree level, and those from Asian backgrounds were more likely to opt for an FP nearer home.

The finding that medical graduates from lower socioeconomic backgrounds, as determined by entitlement to free school meals or having no parent/guardian with a university degree, are more likely to train nearer their parental home resonates with the wider literature on widening access [21, 22, 24–27, 39]. It is perhaps unsurprising that medical students also show the same broad pattern, but this has important implications for the widening participation policies, especially those that impact the socially accountable medical education agenda. Our results re-affirm the assumption that widening participation can boost the number of medical graduates who are more likely to train local and remain in the area to serve their communities [40, 41]. On the other hand, the results also demonstrate how distance may be a deterrent factor for trainees from low social classes in choosing foundation training posts that are far away from parental home. Put another way, it is possible that students from poorer backgrounds are more concerned about moving away from home than their more socially affluent counterparts. Although we did not test that in this study, the reasons for such decisions can vary from financial to socio-cultural factors such as pressure from family, or simply the need to maintain the existing social networks [20, 27, 30]. The association between ethnicity and choice of foundation school can also be linked to previous studies of medical school choice in the UK. For example, Brown’s qualitative study found that Asian female participants indicated the importance of the location of the medical school because of their wish to live at home during their study [42]. However, more qualitative research is needed in order to understand the reasons why certain groups of medical graduates are more likely to choose postgraduate training posts that are closer home.

In this study we have also shown how some regions of foundation schools seem to be more attractive (in terms of being first choice for applicants) than others. Because of the pecking order system, some schools seem to “pull” applicants from elsewhere in the country, which means that other foundation schools/regions “lose” trainees. For example, at the time of this study there was only one medical school in Northern Ireland (NI). This means some people of NI origin do their medical degrees in other UK countries, but may apply to do the foundation programme “back home”. Therefore, given the 92% retention rate of home students, the net gain observed in Table 2 relate to those students who attended medical schools elsewhere but still came back home for postgraduate training. Comparably, Scotland graduates enough medical students to staff its workforce, but those students born outside Scotland mostly plan return to their respective countries for postgraduate training, leaving a significant shortfall of doctors to fill foundation and training posts in Scotland [43].

The London area is the most oversubscribed and largest deanery in the UK. It is responsible for training approximately 20% of all medical trainees, with 11,800 trainees across foundation, core and higher specialty [44]. Despite having a wide catchment area, the region also attracts trainees from all over the country. The reasons for popularity of foundation schools in London area and South of England are readily shared by medical students and trainees in the social media, and these vary from academic reputation, physical environment and location [45]. Our data does not show a complete list of how individual trainees ranked their preferred foundation schools other than the one they were allocated into. However, we can hypothesise that medical graduates who come from affluent backgrounds are ready to compromise the ‘distance from home’ factor and rank low the less favourable places in exchange for ‘pull factors’ of certain foundation schools, regardless of the distance and associated costs. This implies that disadvantage continues for those trainees who do not have the capital to travel far to more popular foundation schools, instead “settling” for ones that are located closer to home.

The finding of this study has implications for both the widening access agenda and equitable distribution of health services. Firstly, the data provides supportive evidence to the case of increased intake of students from widening access backgrounds, whom we have shown are more likely to train locally, thus ultimately becoming an asset for the local workforce. Secondly, medical education and training is a complex system that transcends beyond the borders of UK devolved governments; therefore, understanding the migration pattern by which medical graduates enter the profession could provide useful information for workforce planning, and add voice to the national debate about funding for medical education and training in the devolved nations.

A strength of this study is that we have used the UK Medical Education Database (UKMED) to access a national representative sample of three cohorts of medical graduates from all UK countries. To the best of our knowledge, this is the first study to use the actual travel time to describe the migratory patterns of trainee doctors since the implementation of modernising medical careers. Not least, the use of multilevel modelling is a statistically robust technique that gives assurance that the results are not biased due to non-random effect of geographical location. However, some limitations are worth noting. The foundation programme is only the first step of postgraduate training pathway. Therefore, we do not know how many people want to spend the early years of postgraduate training near home or near their medical school because of familiarity of the place but plan to leave at a later point. It is possible that some trainees will do this as a way of minimising the transition from being a student into being a doctor. However, we did not analyse distance between parental home and medical school. This may be a fruitful focus for future studies. We plan to extend our investigation to examine if the pattern we identified remains the same when doctors apply for core or specialty training. Another limitation of the study is that generalisability may be limited. The data and the observed patterns are only specific to the geography of the UK, and some countries may not have a postgraduate training system that requires trainees to rank postgraduate training options in order of preference. Further research would benefit if the design of this study can be replicated in different country setting.

## Conclusion

In summary, this paper has examined the movement pattern of UK graduates as they transition from medical school to postgraduate training. The majority of the students prefer to train at foundation schools that are reasonably closer to family home, but we have identified that social class differences exist in terms of individual preferences to attend foundation schools that are nearer parental home. We propose that proximity, or lack of proximity, to preferred foundation schools may be a deterrent factor for certain groups of individuals. More research is needed to examine this further.

## Abbreviations

GLMM: Generalised Linear Mixed Models
POLAR: Participation of Local Areas
UKFP: UK Foundation Programme
UKMED: UK Medical Education Database
